# Chorea

**Published:** 1893-12-30

**Authors:** Bertram Rogers

**Affiliations:** Physician to the Hospital for Sick Children and Women, Bristol


### The Hospital Clinic.

[*The Editor will be glad to receive offers of co-operation and contributions from members of the profession. All letters should be addressed to* The Editor, The Lodge, Porchester Square, London, W.]

### CHOREA.

By Bertram Rogers, B.A., M.D., Physician to the Hospital for Sick Children and Women, Bristol.

Few facts are more difficult to explain in medicine than the relationship that exists between certain diseases, and there is no better example of this connection than the three diseases, rheumatism, chorea, and scarlet fever, though how they are related no theory has as yet satisfactorily explained. That they are we see almost daily confirmed, in spite of the fact that in obtaining the history of children's complaints great difficulties beset the path of the physician, for the account has always to come from the parents, who form their own ideas, from which they refuse to retire. Rheumatism, for instance, in children is a disease of much less severity than it is in an adult, and is often overlooked; but in spite of this the proportion of cases of chorea in which a rheumatic history can be obtained is large. We may see a child pass through in a short time an attack of chorea, and then of rheumatism, or *vice versa*, or obtain a history of rheumatism in the family of a patient suffering from chorea, though the actual coincidence of the two diseases is seldom observed, fortunately for the patient.

Chorea, unlike rheumatism, is almost confined to children, it only very rarely attacking adults. Cases, however, do occur at all ages. I have seen an old woman of nearly 80 attacked with it. Pregnant women are occasionally affected by it, and in these cases it appears to become a much more serious disease, for the proportion of deaths from the disease in pregnant women is much higher than it is at any other time. Children of all ages are attacked, but the most susceptible time of life appears to be between the tenth and fifteenth years, and girls suffer from the disease more than boys in about the proportion of two to one. This may be accounted for by the fact that the nervous system of girls is in a more irritable state between these years, when the changes connected with development of womanhood are commencing, and also that the female sex are more liable to disorders of the nervous system at all periods of life, of which we have evidence of the greater frequency of hysteria and emotional attacks in girls. It should not, however, be concluded from this that chorea is a functional disease rather than an organic one; but it is a well-established fact that the sufferers from chorea are often highly neurotic, for we constantly find that a naturally shy and timid girl dates her attack from fright, from threatened punishment, or from the over-pressure brought to bear upon her brain in preparing for an examination. I cannot help believing that the School Board is much too frequently libelled in ascribing to it so many cases of chorea; but over-pressure is the cant phrase of the day, and parents are only too glad to find some shoulders broad enough to bear the blame. We see chorea much more frequently among the poor and ill-fed, but even among the well-to-do we find the disease ascribed to the same causes.

Imitation must, I think, be a rare cause, though well-authenticated cases have been recorded. It is the common practice in all children's hospitals to place choreic patients in the general wards, and we seldom see another patient develop the disease, but there is no doubt that it is advisable not to place two cases side by side, for the sight of one another tends to perpetuate the disease, if not to accentuate it.

The onset is occasionally, but not often, sudden. It is generally noticed that the child makes faces, is unable to button her clothes, to sew or write, and teachers, failing to recognise the cause, by their injudicious treatment tend to increase the disease, by the threat or actual administration of punishment for what is considered by them to be inattention or disrespect, whereas the loss of power, &c., is really only a symptom of the disease.

The disease may be defined as one of the nervous system, characterised by a succession of irregular, clonic, involuntary movements of limited range, occurring in almost all parts of the body.

The movements are characteristically arhythmic and purposeless, and cease during sleep. The upper extremity is the part which suffers most, and in a few mild cases the movements are limited to only one side of the body. A moderately severe case can be recognized at once, the arm is jerked forward and suddenly withdrawn, the fingers move in a purposeless manner, the face is drawn up into queer contortions, and the tongue if protruded is withdrawn with a snap of the jaws. On attempting to stand or walk the legs may join in the movements, but generally to a less extent than the arms or face. Sometimes the respiratory muscles are affected, so that the breathing becomes jerky, and even produces irregularity of the heart. At the onset of the disease the movements are slight, becoming worse in a certain number of cases, but in a few remaining slight all through. It sometimes happens that the clonic movements are so severe and violent that the patient does herself harm by striking her arms and legs against the sides of the bed, and she may wriggle about to such an extent that bedsores may form.

Chorea is not a disease which is cured straight off; it certainly should be classed among the chronic ones, when we remember that its average duration is ten weeks. It does not continue in the same condition all that time, for treatment by drugs and rest in bed, if they do not cure it, very materially lessen the symptoms, but movements may be noticed for months, and relapses or second attacks are not uncommon. Numbers of theories have been propounded to explain these peculiar movements, and not one of them has satisfactorily done so. Pathological changes have been discovered and claimed as the cause, but it is not saying too much to state that they are probably the result of the disease and not the cause. So few cases terminate fatally that post-mortem observations on the state of the brain and cord are not frequent, and in those which do end with the death of the patient it is more often that a complication is answerable for this result than the disease itself. Pathologists in trying to find a cause have not unfrequently mistaken these results of the complication as the starting point of the disease. The probable situation of the morbid process is the cortex of the brain, but in what way the discharge of nerve force originates, or from what cause it starts no satisfactory answer has as yet been given.

The complications most frequently occurring during the disease are of more lasting importance than the disease itself, for while chorea is recovered from completely, and no trace of its presence can be found in after years, it is only too frequently discovered that the patient has to go through life with a disabled mitral valve. Endocarditis is a common complication and a serious one, and just as we find in rheumatism, it is usually the mitral valve that suffers. In a very large proportion of cases a soft systolic murmur develops; sometimes, it is true, due only to anæmia, but too often indicating organic mischief.

The main point about the treatment of chorea is absolute rest in bed with full and sufficient sleep. In fact some physicians have gone so far as to keep their patients continuously asleep for weeks, only allowing them to wake up sufficiently long to be fed and washed. This is, of course, only practised in the severer cases, but the importance of a due amount of sleep is great.

Various drugs have been employed to quiet the movements of the patient, and the universal favourite is arsenic. It is generally given in small doses, but I have seen excellent results follow the administration of full doses, reduced when the patient is quieter. Large doses of conium will quiet the movements, but the drug is not one which can be persevered in for any length of time. Other drugs employed are sulphate of zinc, cannabis indica, bromides, chloral, calabar bean, and hyoscyamine. All of these have their advocates, but have not found universal favour. Some physicians go so far as not to give any drugs at all, but rely on rest and general treatment. The diet should be generous seeing that the patients are mostly underfed and anæmic, and the bowels should be regulated, as constipation certainly appears to aggravate the disease. We have no drug that will cut short endocarditis.

